# A de novo start‐lost variant in *ANKRD28* in a Holstein calf with dwarfism

**DOI:** 10.1111/age.13204

**Published:** 2022-04-22

**Authors:** Joana G. P. Jacinto, Irene M. Häfliger, Christine F. Baes, Hinayah R. de Oliveira, Cord Drögemüller

**Affiliations:** ^1^ 9296 Department of Veterinary Medical Sciences University of Bologna Ozzano Emilia (Bologna) Italy; ^2^ 27210 Vetsuisse Faculty Institute of Genetics University of Bern Bern Switzerland; ^3^ 3653 Department of Animal Biosciences Centre for the Genetic Improvement of Livestock University of Guelph Guelph Ontario Canada

## BACKGROUND

Osteochondrodysplasias comprise a broad spectrum of hereditary developmental disorders of bone and cartilage, but can also have significant effects on tendons, ligaments, and muscles (Warman et al., 2011). According to Online Mendelian Inheritance in Animals, there are six known dwarfism phenotypes in cattle, which are inherited both recessively and dominantly and are due to structural or signaling pathways disruptions (OMIA 000299‐9913; Boegheim et al., 2017).

## ANALYSIS

A 3.5‐month‐old, female, purebred Holstein calf was reported because of its short stature, protruding scapulohumeral joints, increased concavity of the frontal bone, and bossing of the intercornual prominence (Figure S1). A whole‐genome sequencing (WGS) approach was performed as described before (Jacinto et al., 2022) using genomic DNA extracted from blood of the affected calf, her dam, and from semen of her sire. Reads were mapped to the ARS‐UCD1.2 assembly (Rosen et al., 2020) resulting in an average read depth of 20.7× in the calf, 21.8× in the dam, and 22.1× in the sire, and then processed as reported earlier (Jacinto et al., 2021). We hypothesized that the affected calf had a novel form of dwarfism and investigated the genetic origin. The trio‐based WGS approach identified no homozygous protein‐changing variants present exclusively in the genome of the affected calf. Consequently, a recessive inheritance seems unlikely. Given that the reported calf was the only case and dwarfism had never before been reported in the Canadian Holstein population, we hypothesized that a dominant mode of inheritance due to a de novo mutation event was more likely. We found three heterozygous private protein‐changing variants present in the calf and absent in both parental genomes and in 5365 controls (Table S1). Review of the sequencing data for each of these heterozygous variants revealed that all were indeed de novo variants affecting different genes. Only one of these variants concerns an interesting putative candidate gene (*ANKRD28*) for the observed phenotype. This heterozygous variant at chr1:152807533C>CA represents a 1‐bp insertion in exon 2 of the *ANKRD28* gene that directly affect the translation initiation (start) codon (XM_024989836.1: c.2dupT; Figure S1). The predicted consequence of this start‐lost variant is that amino acid Met1 is converted to Leu after activation of an upstream translation initiation site at cDNA position −20, resulting in N‐terminal protein extension by insertion of six amino acids between Met1 and Gly2 (XP_024845604.1: p.Met1_Gly2insIleValGlyGlyLysAlaLeu).

## COMMENTS

We propose the heterozygous c.2dupT variant as a candidate causative variant for the observed congenital disorder and *ANKRD28* as a novel candidate gene for dwarfism phenotypes. The protein‐altering nature of this de novo mutation strongly suggests the causality of the variant. The Genome Aggregation Database showed that *ANKRD28* falls into the class of loss‐of‐function haploinsufficient genes (Karczewski et al., 2020). Therefore, two different situations could explain the perceived phenotype: either haploinsufficiency or co‐expression of an N‐terminally extended protein, leading to non‐ or abnormal expression of the mutant allele.

Nonetheless, since this is a single case study and we have no functional confirmation, this result must be considered preliminary and should be interpreted with caution. It must also be noted that the analysis of short‐read genomic data is not ideal for identifying larger structural variants. Further individual cases of dwarfism in cattle or other animal species could be investigated for *ANKRD28* variants by DNA sequencing.

## CONFLICT OF INTEREST

The authors declare no conflict of interest.

## Supporting information

Supplementary Material

## Data Availability

The WGS data are available under the study accession no. PRJEB18113 at the European Nucleotide Archive (www.ebi.ac.uk/ena; calf SAMEA8565094, dam SAMEA8565093, sire SAMEA8565095).
