# Supplementary material for: A de novo start‐lost variant in ANKRD28 in a Holstein calf with dwarfism
Source: Anim Genet. 2022 Apr 22;53(3):470–1. doi: 10.1111/age.13204 (PMC9373846; doi:10.1111/age.13204)
Supplement: Supplementary file 1 — Supplementary Material [file AGE-53-470-s001.docx]

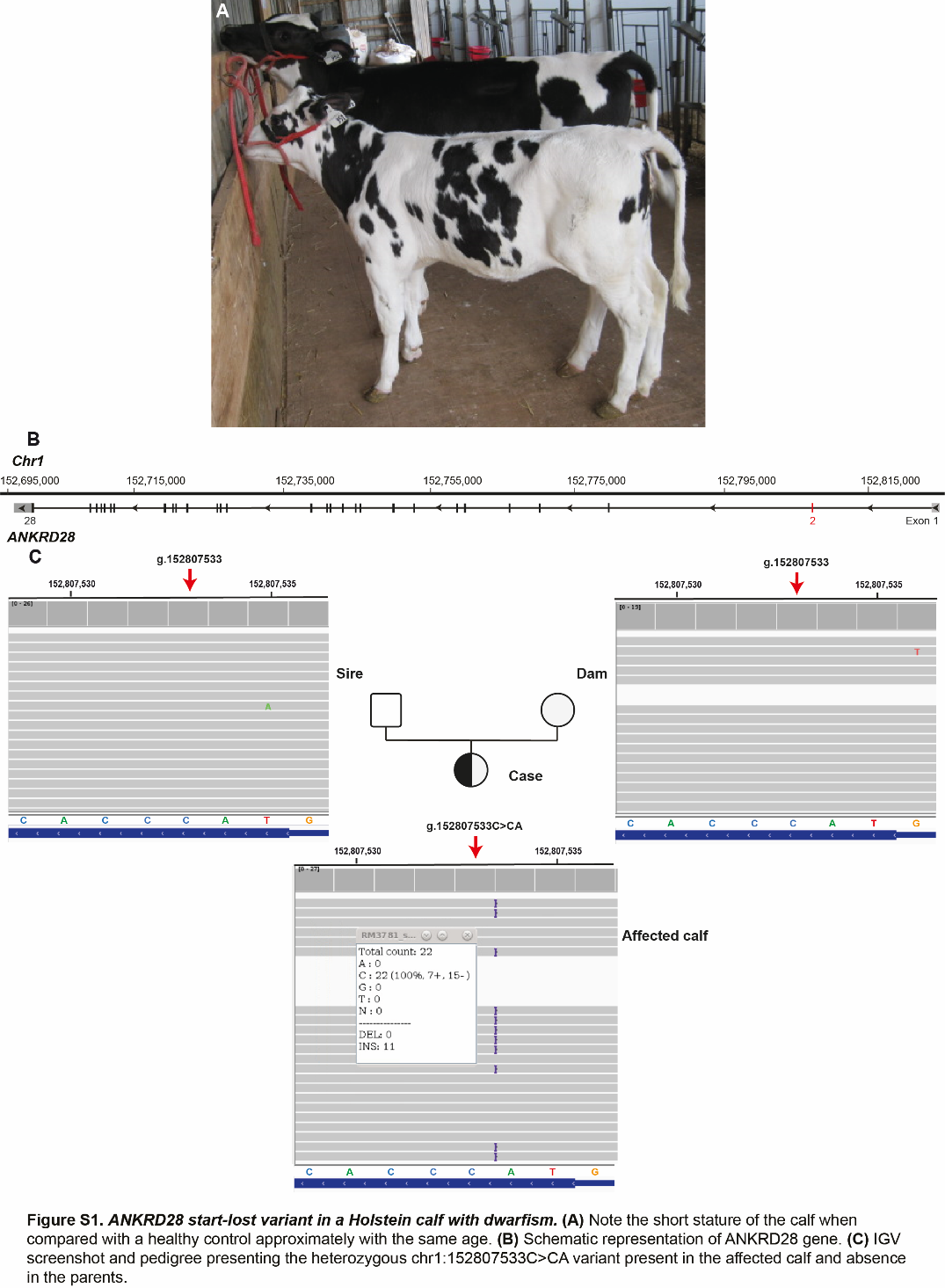
**Figure S1.** *ANKRD28* frameshift insertion in a Holstein calf with dwarfism.

(A) Note the short stature of the calf when compared with a healthy control approximately with the same age. (B) Schematic representation of *ANKRD28* gene*.* (C) IGV screenshot and pedigree presenting the chr1:152807533C>CA variant in present in the affected calf and absence in the parents.

**Table S1** List of private heterozygous variants in the affected calf.

This list was obtained after the comparison of 5365 cattle genomes of different breeds sequenced as part of the ongoing Swiss Comparative Bovine Resequencing project (<https://www.ebi.ac.uk/ena/browser/view/PRJEB18113>) and Run 9 of the 1000 Bull Genomes Project (Hayes and Daetwyler, 2019) and after visual inspection with Integrative Genomes Viewer software (Robinson et al., 2017), revealing 3 protein-changing variants with a predicted moderate or high impact.

Hayes, B. J. and Daetwyler, H. D. (2019) ‘1000 Bull Genomes Project to map simple and complex genetic traits in cattle: Applications and outcomes’, Annual Review of Animal Biosciences, 7, pp. 89–102. doi: 10.1146/annurev-animal-020518-115024.

Robinson, J. T. et al. (2017) ‘Variant review with the integrative genomics viewer’, Cancer Research, 77(21), pp. e31–e34. doi: 10.1158/0008-5472.CAN-17-0337.

| **#CHROM** | **POS** | **REF** | **ALT** | **EFFECT** | **IMPACT** | **GENE** | **Associated disorder/ gene function** | **GENEID** | **OMIM** | **FEATURE** | **FEATUREID** | **BIOTYPE** | **RANK** | **HGVS_C** | **HGVS_P** | **Provean score** | **Provean predicted impact** |
| --- | --- | --- | --- | --- | --- | --- | --- | --- | --- | --- | --- | --- | --- | --- | --- | --- | --- |
| 1 | 152807533 | C | CA | frameshift_variant&start_lost | HIGH | ANKRD28^1^ | Involved in the recognition of phosphoprotein substrates | 529062 | 611122 | transcript | XM_024989836.1 | protein_coding | 2 | c.2dupT | p.Met1fs | NA | NA |
| 17 | 65054770 | G | C | missense_variant | MODERATE | SGSM1^2^ | Promotes GTP hydrolysis by RAB34 and RAB36 | 515653 | 611417 | transcript | NM_001192421.1 | protein_coding | 12 | c.1355G>C | p.Arg452Thr | -0.467 | Neutral |
| 23 | 9284981 | T | G | missense_variant | MODERATE | DEF6^3^ | Immunodeficiency 87 and autoimmunity | 516457 | 610094 | transcript | NM_001098994.1 | protein_coding | 4 | c.458T>G | p.Met153Arg | -4.182 | Deleterious |

^1^ The *ANKRD28* gene encodes serine/threonine-protein phosphatase 6 regulatory ankyrin repeat subunit A, which contains 26 ankytrin repeats that mediate protein-protein interactions (Mosavi *et al.*, 2004). In addition, ANKRD28 is an important binding partner of DOCK180 that promotes cell migration (a fundamental event during early development) by regulating the formation of focal adhesions and specifying the localization and activity of the DOCK180-Rac1 pathway (Tachibana *et al.*, 2009). It could be speculated that the predicted consequence of the start-lost variant altering the beginning of the ANKRD28 protein might disrupt the DOCK180-Rac1 pathway. Interestingly, the absence of *DOCK180* in mice leads to musculoskeletal disorders (Laurin *et al.*, 2008).

Laurin, M. et al. (2008) ‘The atypical Rac activator Dock180 (Dock1) regulates myoblast fusion in vivo.’, Proceedings of the National Academy of Sciences of the United States of America, 105(40), pp. 15446–15451. doi: 10.1073/pnas.0805546105.

Mosavi, L. K. et al. (2004) ‘The ankyrin repeat as molecular architecture for protein recognition.’, Protein science : a publication of the Protein Society, 13(6), pp. 1435–1448. doi: 10.1110/ps.03554604.

Tachibana, M. et al. (2009) ‘Ankyrin repeat domain 28 (ANKRD28), a novel binding partner of DOCK180, promotes cell migration by regulating focal adhesion formation.’, Experimental cell research. United States, 315(5), pp. 863–876. doi: 10.1016/j.yexcr.2008.12.005.

^2^ The effect of the missense variant in the *SGSM1* gene was predicted to be neutral.

^3^ The amino acid substitution in *DEF6* was found to be deleterious. Pathogenic variants of human *DEF6* are associated with a recessively inherited form of immunodeficiency (MIM 610094), a hereditary disease that has no parallels to the phenotype presented here, and the affected calf is only a heterozygous carrier.
